# Retraction: Carbon black hybrid material furnished monodisperse platinum nanoparticles as highly efficient and reusable electrocatalysts for formic acid electro-oxidation

**DOI:** 10.1039/d1ra90153b

**Published:** 2021-10-12

**Authors:** Laura Fisher

**Affiliations:** Royal Society of Chemistry Thomas Graham House, Science Park, Milton Road Cambridge UK advances-rsc@rsc.org

## Abstract

Retraction of ‘Carbon black hybrid material furnished monodisperse platinum nanoparticles as highly efficient and reusable electrocatalysts for formic acid electro-oxidation’ by Yunus Yıldız *et al.*, *RSC Adv.*, 2016, **6**, 32858–32862, DOI: 10.1039/C6RA00232C

The Royal Society of Chemistry hereby wholly retracts this *RSC Advances* article due to concerns with the reliability of the data in the published article.

The high resolution transmission electron micrograph inset in Fig. 2 that represents Pt NPs/TPrA@VC-AC is a duplicated and rotated version of the inset in Fig. 2a in an *International Journal of Hydrogen Energy* article by the same authors,^1^ which is a high resolution transmission electron micrograph representing Pt/TPA@rGO NPs.

Fig. S1 in the ESI of this *RSC Advances* article, which is a transmission electron micrograph representing Pt NPs/TPrA@VC, is a duplicated and scaled version of Fig. S2, also in the ESI, which is a transmission electron micrograph representing Pt NPs/TPrA@AC.

The authors claim that these are mistakes and provided replacement data for consideration. However, an expert reviewed the authors’ response and concluded that it did not satisfactorily address the concerns, and that the replacement figures did not fully support the conclusions. Given the significance of the concerns about the validity of the data, the findings presented in this paper are no longer reliable.

Fatih Sen opposes this retraction. Handan Pamuk, Yunus Yıldız, Ozlem Karatepe and Zeynep Dasdelen were contacted but did not respond.

Signed: Laura Fisher, Executive Editor, *RSC Advances*

Date: 23rd September 2021

## Supplementary Material
